# A redescending M-estimator approach for outlier-resilient modeling

**DOI:** 10.1038/s41598-024-57906-1

**Published:** 2024-03-26

**Authors:** Aamir Raza, Muhammad Noor-ul-Amin, Amel Ayari-Akkari, Muhammad Nabi, Muhammad Usman Aslam

**Affiliations:** 1 Govt. College Women University Sialkot, Sialkot, Pakistan; 2 Lahore Campus, COMSATS University Islamabad, Lahore, Pakistan; 3 Biology Department, College of Sciences in Abha, King Khalid University, P.O. Box 960, Abha, Saudi Arabia; 4 Khost Mechanics Institute, Khost, Afghanistan; 5 Xi’an Jiaotong University, Xi’an, China

**Keywords:** Ordinary least squares, Outliers, Redescending, Robust regression, Energy science and technology, Engineering, Materials science, Mathematics and computing

## Abstract

The OLS model is built on the assumption of normality in the distribution of error terms. However, this assumption can be easily violated, especially when there are outliers in the data. A single outlier can disrupt the normality assumption of error terms, making the OLS model less effective. In such situations, M-estimators (MEs) come into play to obtain reliable estimates. We introduce a redescending M-estimators (RME) for robust regression to handle datasets with outliers. The proposed RME produces more robust estimates by effectively managing the influence of outliers, even at lower values of the tuning constant. We compared the performance of this estimator with existing RMEs using real-life data examples and an extensive simulation study. The results show that our suggested RME is more efficient than the compared ME in various situations.

## Introduction

The ordinary least square (OLS) method has many useful applications in real life, but sometimes real data do not fulfill the conditions of OLS in terms of normality of error terms due to the presence of outliers. Even a single value of the outlier can disturb the performance of the least square estimates by producing inefficient and unreliable results. To overcome this drawback of OLS, researchers developed a technique called robust regression, which modifies the OLS in the presence of outliers. In heavy tailed distributions, the OLS estimates are badly influenced by the presence of outliers and MEs provide a possible alternative to the classical OLS estimates to compensate the sensitivity of the estimates towards the outliers. A large number of robust estimators are presented in the literature: M-estimators (Huber^1^), least median square (Roisseeuw^2^), least trimmed square (Roisseeuw^3^), MM estimators (Yohai^4^) and S-estimators (Sakata and White^5^) are very famous robust estimators.

Huber^1^ introduced the procedure of the ME to deal with the data having outliers. This estimator provided weights approximately equal to one for the central observations and zero weights for outliers. The maximum likelihood formulation is used in ME when the condition of normality in error terms is violated in OLS. In robust MEs, the symmetric loss function is used in place of the square error term in OLS, i.e.

1 $$\mathop {Minimize}\limits_{{\hat{\beta }}} \sum\limits_{k = 1}^{n} {\rho \left( {r_{k} } \right)}$$

where $\rho \left( . \right)$ provides the role of error terms in the objective function. The technique of the ME is usually based on the iterative reweighted least squares technique (IRLS) to obtain the optimum estimates of objective function, Birch^6^. The objective function is chosen in such a way that it reduces the weight of outliers and hence produces maximum efficiency.

The psi function is obtained by differentiating Eq. (1) w.r.t. residual, the $\psi \left( {r_{k} } \right)$ function is given as.

2 $$\sum\limits_{k = 1}^{n} {\psi \left( {r_{k} } \right)} X_{k} = 0$$

Weight function $w\left( {r_{k} } \right)$ is obtained by dividing $\psi \left( {r_{k} } \right)$ function by “*r*” residuals, given as

3 $$\sum\limits_{k = 1}^{n} {w\left( {r_{k} } \right)} X_{k} = 0$$

If the deviation of $\rho \left( . \right)$ is redescended, the ME is known as the RME. Many researchers have done useful work on ME; well-known names are Hample (1986), Andrew^7^, Tukey^8^, Qadir^9^, Ali (2005), Insha^10^, Alamgir (2015), Khalil (2016), Noor-ul-Amin^11^, Anekwe^12^, Luo^13^, and Mukhtar^14^. These RME perform well to reduce the effects of outliers. We have also proposed a RME to deal with the data having outliers given in Eq. (13). The proposed estimator is a generalized RME, as the weights of outliers can be controlled by changing the value of the generalized tuning constant ‘*a*’.

The design of the upcoming sections is as follows: Section “Redescending M-estimator” describes the existing RME; Section “Redescending M-estimator” discusses the proposed RME with its graphical presentations. In Section “Proposed redescending M-estimator”, the performance of the proposed ME relative to existing ME is presented. In Section “Graphical comparison of the proposed RME with existing RME”, practical applications and a simulation study of the proposed RME are discussed in Session 6. The concluding remarks of the proposed ME are cited in Section “Simulation study”.

## Redescending M-estimator

A detailed discussion about the existing ME is necessary to highlight the superiority of the proposed estimator. So we have done a detailed study on the existing ME in this section. The known MEs are given below.

Huber^1^ proposed well known ME with $\psi$ function defined as

4 $$\psi \left( r \right) = \left\{ {\left\{ {\begin{array}{*{20}c} r & {\left| r \right| < k} \\ k & {\left| r \right| \ge k} \\ \end{array} } \right.} \right\}$$

where *k* is the tuning constant and *r* is the residual obtained using the OLS. The value of *k* is 1.345 to obtain 95% efficient result for the data having Gaussian distribution. The efficiency of Huber proposed model is found to be low when data contain larger residuals as its ME has not redescending characteristics.

Hampel (1986) introduced a piece wise ME with-function given by

5 $$\psi \left( r \right) = \left\{ {\begin{array}{*{20}l} r \hfill & {\left| r \right| \le k} \hfill \\ {k.sign(r)} \hfill & {k < \left| r \right| \le l} \hfill \\ {k\frac{m - \left| r \right|}{{m - l}}sign(r)} \hfill & {l < \left| r \right| \le m} \hfill \\ 0 \hfill & {\left| r \right| > m} \hfill \\ \end{array} } \right\}$$

where *k, l* and *m* are constant and $0 < k \le l < m < \infty$. Differentiability of $\psi \left( r \right)$ is not ideal here and a smooth $\psi \left( r \right)$ function should be preferred. This suggested robust estimator performed well for the Princeton Robustness Study. The Hampel psi function has lack of differentiability, and it uses three tuning constants, which are undesirable.

Andrew^7^ developed a very popular and commonly used robust ME called the Andrew sine function,$\psi$-function for Princeton Robustness Study is written as

6 $$\psi \left( r \right) = \left\{ {\begin{array}{*{20}l} {k\sin (r/k)} \hfill & {\left| r \right| < k\pi } \hfill \\ 0 \hfill & {\left| r \right| > \pi k} \hfill \\ \end{array} } \right\}$$

where *k* is a tuning constant and the value of *k* = 3.2 to obtain maximum efficiency. This function is smooth and differentiable.

Another very useful and popular redescending robust estimator is given by Tukey^8^ with $\psi$-function defined as

7 $$\psi \left( r \right) = \left\{ {\begin{array}{*{20}l} {r\left[ {1 - \left( \frac{r}{k} \right)^{2} } \right]^{2} } \hfill & {\left| r \right| < k} \hfill \\ 0 \hfill & {\left| r \right| > k} \hfill \\ \end{array} } \right\}$$

where *k* is the tuning constant. For Tukey’s ME, value of *k* = 4.865 to obtain 95% efficiency for the data have Gaussian distribution. Tukey’s bi-weighted and Andrew’s sine functions covers the drawbacks of Hampel  $\psi$ function to some extent, but they give less weight to some good observations.

Qadir^9^ developed another RME known as the Qadir Beta function. The $\psi$-function is given as

8 $$\psi \left( r \right) = \left\{ {\begin{array}{*{20}l} {\frac{r}{{16k^{4} }}\left( {k + r} \right)^{2} \left( {k - r} \right)^{2} } \hfill & {\left| r \right| \le k} \hfill \\ 0 \hfill & {\left| r \right| > k} \hfill \\ \end{array} } \right\}$$

where *k* is an arbitrary constant called the tuning constant, the value of *k* is 4.0 to attain maximum efficiency.

Ali (2005) also derived a modified Tukey’s RME with the psi function.

9 $$\psi \left( r \right) = \left\{ {\begin{array}{*{20}l} {\frac{2r}{3}\left[ {1 - \left( \frac{r}{k} \right)^{4} } \right]^{2} } \hfill & {\left| r \right| \le k} \hfill \\ 0 \hfill & {\left| r \right| > k} \hfill \\ \end{array} } \right\}$$

This ME gives most efficient results if *k* = 4.

Insha^10^ developed a RME for the detection of outliers, whose $\psi$-function is given as

10 $$\psi \left( r \right) = \left\{ {\begin{array}{*{20}c} {\left[ {1 + \left( \frac{r}{k} \right)^{4} } \right]^{ - 2} } & {\left| r \right| \ge 0} \\ \end{array} } \right\}$$

Tuning constant *k* = 4 provides the most efficient results for Insha’s robust estimator.

Alamgir (2015) suggested another RME for robust regression; his proposed psi-function is given as

11 $$\psi \left( r \right) = \left\{ {\begin{array}{*{20}l} {\frac{{16r.e^{{ - 2\left( {r/k} \right)^{2} }} }}{{\left( {1 + e^{{ - \left( {r/k} \right)^{2} }} } \right)^{2} }}} \hfill & {\left| r \right| \le k} \hfill \\ 0 \hfill & {\left| r \right| > k} \hfill \\ \end{array} } \right\}$$

Alamgir suggested *k*=3 to obtain 95% efficient estimates for the data having a normal distribution. In the series of RME developments, Khalil (2016) also suggested his RME, whose psi-function is given as

12 $$\psi \left( r \right) = \left\{ {\begin{array}{*{20}l} {r\left( \frac{3}{2} \right)\left\{ {1 - \left( \frac{r}{k} \right)^{4} } \right\}^{2} \sin \left[ {\left( \frac{2}{3} \right)\left\{ {1 - \left( \frac{r}{k} \right)^{4} } \right\}^{2} } \right]} \hfill & {\left| r \right| \le k} \hfill \\ 0 \hfill & 0 \hfill \\ \end{array} } \right\}$$

where *k* is the tuning constant, and a value of *k* = 4 is suggested by Khalil to obtain 95% efficiency in the normal case. The ME proposed by Ali, Alamgir, and Khalil rejected the observations completely with a larger residual. Insha provided an estimator that covers this drawback, but it lacks generalization. We have produced a RME that covers the drawbacks of previously proposed estimators and has redescending characteristics. If the outliers are completely rejected by the ME, then it is called a RME. Hence, a ME is redescending if the derivative of the objective function, i.e.$\rho \left( {r_{k} } \right)$ is redescending that satisfies $\mathop {\rho^{\prime}\left( {r_{k} } \right)}\limits_{{r_{k} \to \pm \infty }} = 0$.

## Proposed redescending M-estimator

We proposed a new RME called Aamir’s RME, which contains the properties of a redescending estimator for the detection of outliers in robust regression. The proposed RME is more robust even at low value of tuning constant, it produces more efficient and reliable estimate even at small sample sizes, the mathematical formulation of the suggested RME is simple and easy to apply in real life situations, the proposed RME is generalized ME as we can adjust the weights of extreme values of residuals by changing the value of the generalized tuning constant ‘*a’*. The psi function of proposed estimator is more linear than the existing RMEs. The proposed estimator is more flexible to control the weights of outliers. The validity and usefulness of the proposed estimator are confirmed by extensive simulation analysis. The characteristics and shape of $\rho \left( {r_{i} } \right)$ function, $\psi \left( {r_{i} } \right)$ and $w\left( {r_{i} } \right)$ weight function of proposed RME are discussed below.

The objective function of proposed ME is given as

13 $$\rho \left( {r_{i} } \right) = \left\{ {\begin{array}{*{20}c} {\frac{{k^{2} }}{2a}\left[ {1 - \left\{ {1 + \left( \frac{r}{k} \right)^{2} } \right\}^{ - a} } \right]} & {\left| r \right| \ge 0} \\ \end{array} } \right\}$$

where *k* is any arbitrary tuning constant and ‘*a’* is a generalized tuning constant, and we have taken values of ‘*a’* is 6 and 8 for our present study, and *'r'* is the residual associated with i^th^ observation. The proposed objective function fulfills the following properties of $\rho \left( . \right)$ function.

(i) $$\rho \left( {r_{i} } \right) \ge 0$$
(ii) $$\rho \left( 0 \right) = 0$$
(iii) $$\rho \left( { - r_{i} } \right) = \rho \left( {r_{i} } \right)$$
(iv) $\rho \left( {r_{i} } \right) \ge \rho \left( {r_{j} } \right)\,for\,\left| {r_{i} } \right| \ge \left| {r_{j} } \right|$, i.e. $\rho \left( . \right)$ is an increasing function
(v) $\rho \left( . \right)$ is continuous and differentiable

**Figure 1:**
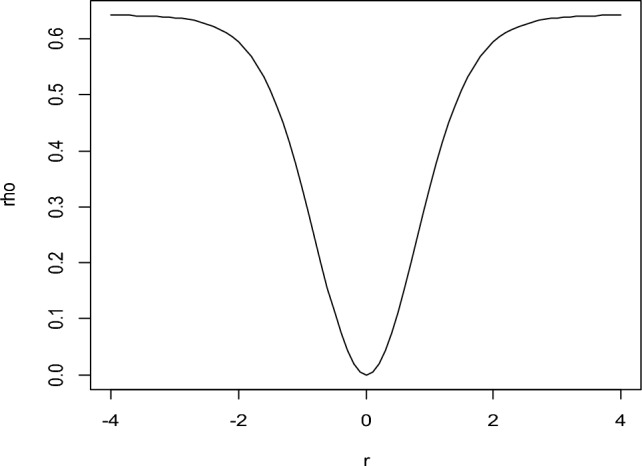
The Graph of $\rho \left( {r_{i} } \right)$ Function of Proposed RME w.r.t. Residual (r).

To demonstrate the performance of the proposed objective function, a sequence of residuals is generated using the R program. Unitizing the generated data, the graph of the objective function given in Eq. (13) is constructed and presented in Fig. 1.

Figure 1, shows that the proposed function is a positive, symmetrical, differentiable, and increasing function which satisfies the conditions of the objection function for robust regression.

Differentiating $\rho \left( . \right)$ function with respect to residual, we obtained $\psi$- function, which is given below

14 $$\psi \left( {r_{i} } \right) = \left\{ {\begin{array}{*{20}c} {r\left[ {1 + \left( \frac{r}{k} \right)^{2} } \right]^{ - a - 1} } & {\left| r \right| \ge 0} \\ \end{array} } \right\}$$

**Figure 2:**
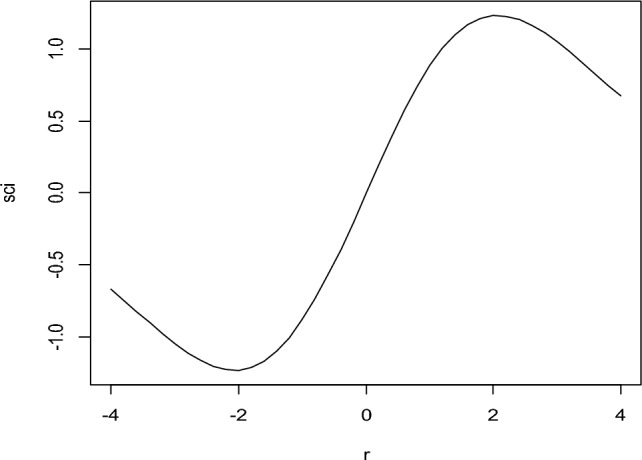
Graph of $\psi$-function for proposed RME w.r.t. residual (r).

Data used in Fig. 1, the graph of psi function is constructed and shown in Fig. 2.

Figure 2 shows that the proposed function is differentiable and more linear at the center than the other existing $\psi$-function of robust estimators. The proposed $\psi$-function follows the pattern needed to construct the RME. Moreover, the proposed $\psi$-function provides more weightage to the central values and vice versa.

Dividing the $\psi$-function by residual “*r*”, we obtain the corresponding weight function, which is given as

15 $$w\left( {r_{i} } \right) = \left\{ {\begin{array}{*{20}c} {\left[ {1 + \left( \frac{r}{k} \right)^{2} } \right]^{ - a - 1} } & {\left| r \right| \ge 0} \\ \end{array} } \right\}$$

**Figure 3:**
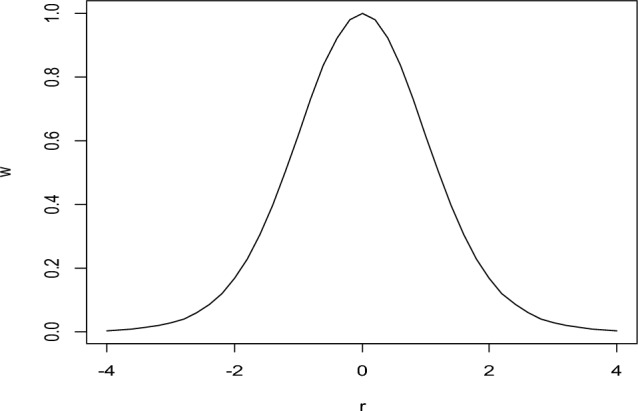
Graph of Weight Function of Proposed RME w.r.t. Residual (r).

Graph of weight function using the data in Fig. 1, is represented in Fig. 3.

Figure 3, shows that the proposed weight function has the unique property of robustness with a generalized tuning constant '*a*'. By increasing the value of ‘*a*’, we can obtain more redescending estimates. We can control the weights of outliers by selecting the value of ‘*a*’. it is analyzed that if the value of the generalized constant is higher, the proposed weight function provides lower the weights of the outliers, and vice versa.

## Graphical comparison of the proposed RME with existing RME

**Figure 4:**
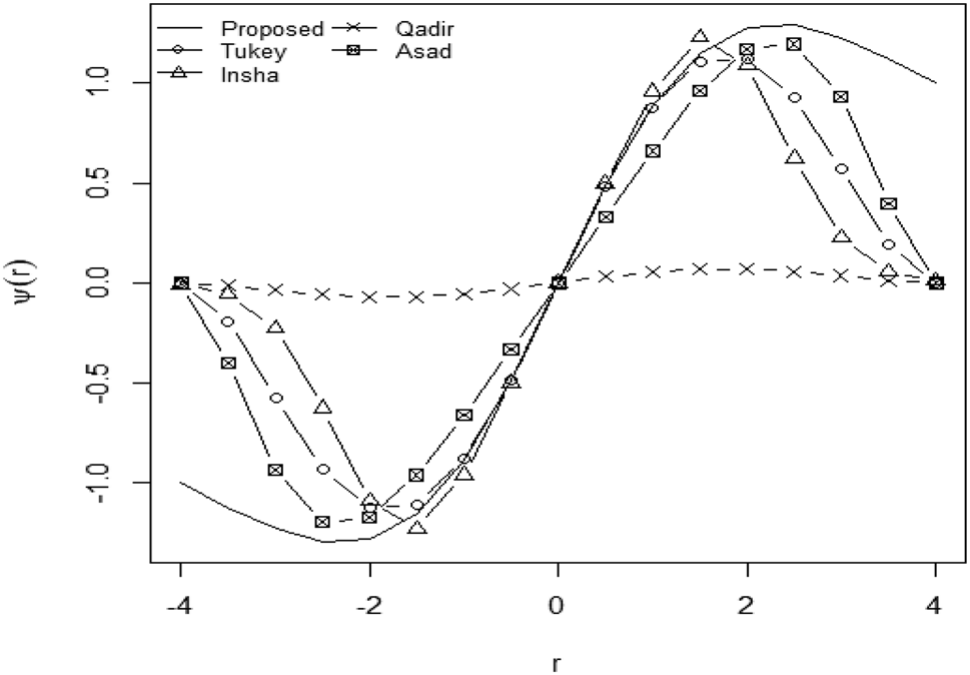
Graphical Comparison of Proposed $\psi$-Function with Well-known $\psi$-Functions w.r.t. Residual (r).

The comparison between the existing RME and the proposed estimator is done by using a graphical representation of $\psi$-functions. The proposed function is continuous differentiable at every point, and it’s $\psi$-function has more linearity at the center of the data than the other existing redescending $\psi$–functions which enhances the efficiency of suggested ME. The graphical comparison of existing and proposed -functions is represented in Fig. 4.

Figure 4 indicates that the suggested psi function is much more linear at the center and more continuous than all previously suggested RME. For example, psi function given by Asad Ali gives a weight of approximately one to the central values, but weighted values rapidly tend to zero for the lager residuals. The process of weighting the outliers approaches zero more rapidly for the estimators proposed by Tukey, Insha, and Qadir, respectively, as compared to the proposed psi function. Hence, the proposed RME is more useful and efficient than all existing ME.

## Practical applications

Two real-life data examples and extensive simulation studies are included in this section to demonstrate the performance of the proposed ME. The proposed RME is found to be more efficient than considered estimators.

### Example-1: number of phone calls from Belgium 1950–1973

The first example is taken from the article by Rousseeuw^3^, which represents the year-wise number of international calls (in tens of millions) from Belgium 1950–1973 (Belgium Statistical Survey) given in Table 1. This data has some outliers in the response variable. The years represent the independent variable X, and the numbers of annual calls represent the dependent variable Y. Some of the authors, Qadir^9^, Ali (2005), and Khalil (2016), have used this in their research. The performance of the proposed RME with the considered estimators is presented in Table 2.

**Table 1:** Data Set of Number of International Calls (in tens of millions) from Belgium.

Years	Calls	Years	Calls
1950	0.44	1962	1.61
1951	0.47	1963	2.12
1952	0.47	1964	11.9
1953	0.59	1965	12.4
1954	0.66	1966	14.2
1955	0.73	1967	15.9
1956	0.81	1968	18.2
1957	0.88	1969	21.2
1958	1.06	1970	4.3
1959	1.2	1971	2.4
1960	1.35	1972	2.7
1961	1.49	1973	2.9

**Table 2:** Fitting OLS, Existing M-Robust Methods and Proposed RME on Telephone Call Data.

Method	Coefficients			
	*Constant*	*X*	Values used	*SSE*
OLS	− 26.01	0.504	24	695.44
LMS	− 5.164	0.108	16	0.1313
Andrew (1.5)	− 4.9072	0.1033	16	0.17308
Tukey (3.8)	− 5.2439	0.1102	16	0.13665
Qadir (1.0)	− 5.505	0.1122	16	0.36726
Ali (3.0)	− 5.2092	0.1094	16	0.13404
Insha (4)	− 5.243	0.1102	16	0.1406
Alamgir (3.0)	− 5.2454	0.1102	16	0.14127
Khalil (4.0)	− 5.2343	0.1099	16	0.13863
Proposed (a = 6, k = 3.5)	− 5.5109	0.1094	16	0.13256

Table 2 depicts the performance of OLS and other robust estimators along with our proposed robust estimator in terms of estimates of regression coefficient and sum of squares of errors (SSE). The OLS estimates are very poor and misleading everywhere within the data due to the presence of outliers with the highest SSE, i.e., 659.44. All the remaining ME perform efficiently to minimize the effect of outliers, but our proposed robust estimator has the least SSE among all except the LMS estimator. So it is analyzed that the suggested estimator outfits the model by giving the least SSE, like the LMS method.

### Example-2: annul average price growth rate in China 1940 to 1948

The data used in this example is taken from Rousseeuw^3^. Insha^10^ also used this data in his research work on robust regression. This data consists of nine values of the annual average growth rates of prices from 1940 to 1948. The response variable is represented by the annual average growth rate, where years represent the predictor. The growth rates are 1.62, 1.63, 1.90, 2.64, 2.05, 2.13, 1.94, 15.50, and 364.00. It means growth rates increased by 1.62% in 1940 as compared with 1939, 1.63% in 1941 as compared with 1940, and so on. An exponential growth in prices was seen in 1948 due to a budget deficit, the war, and massive government expenses. The last two values of the response variable are outliers, as they are very different from other values. The performance of the proposed RME with considered estimators is presented in Table 3.

**Table 3:** Fitting OLS, Existing M-Robust Methods and Proposed RME on Annul Average Price Growth Rate in China 1940–1948.

Method	Coefficients			
	*Constant*	*X*	Values used	*SSE*
OLS	− 1049	24.85	9	78,532.88
LMS	− 2.47	0.102	7	0.69534
Andrew (0.58)	− 2.7542	0.10898	7	0.616687
Tukey (2)	− 2.7535	0.10896	7	0.616652
Qadir (1.0)	− 2.7779	0.1095	7	0.617785
Ali (3.0)	− 2.7792	0.10956	7	0.617851
Insha (1.5)	− 2.6392	0.10618	7	0.615962
Alamgir (3.0)	2.7853	0.1097	7	0.61815
Khalil (2.0)	− 2.7851	0.1097	7	0.618139
Proposed (a = 8, k = 2)	− 2.6486	0.10656	7	0.611112

The OLS method is applied along with other robust estimators, including the proposed robust method, on the average growth rate increase in China since 1940–1948 to check the relative performance of the proposed method with existing robust methods. We have calculated the regression coefficients and SSEs for all methods in Table 3 using R-program. A critical analysis of the results obtained shows that the performance of OLS is very poor and goes astray throughout the data. It also depicts the sensitivity of OLS towards the outliers, and the SSE is highest for the OLS method, which is 78,532.88. All remaining robust estimators perform well to tackle the effects of outliers, but among all methods, our proposed robust estimator has a minimum SSE. This means our proposed estimator is superior among all the estimators to deal with the data having outliers. One can also use real life data set included by Yasin (2021) from the Economic Survey of Pakistan 2017–18.

## Simulation study

The validation and reliability of the proposed ME are done by using an extensive simulation study. The efficiency of the proposed RME is compared with well-known RME. We have used the following linear model for simulation

$$y_{i} = \alpha + \beta x_{i} + e_{i}$$

where $\alpha = 2$,$\beta = 1$, $X_{i} \sim N\left( {20,10} \right)$ and $e_{i} \sim N\left( {0,1} \right)$. We have generated a population of 10,000 values by using the above model. A sample of 100 values is chosen from the population using the R-Program, and estimates of parameters are obtained using the proposed and considered MEs, results are shown in Table 4.

**Table 4:** Coefficient Estimates using Different RMEs for *n* = *100.*

Method	*Case-I*		*Case-II*	
	*a*	*b*	*a*	*b*
OLS	1.99842	1.00002	7.0426	0.99794
Hample (4)	1.99742	1.00008	1.99586	1.00018
Tukye (4.69)	1.99742	1.00011	1.99465	1.00023
Andrew(3.2)	1.99801	1.00021	1.99484	1.00045
Huber (4)	1.99834	1.00002	2.1976	0.99764
Insha (4)	2.00924	0.99964	1.99743	1.00004
Ali (4)	2.00303	0.99984	1.99923	1.00011
Khalil (5)	2.02813	0.99846	2.00358	0.999761
Proposed (2)	1.99736	1.00009	1.99331	1.00047

We have studied two cases; in the first case, we estimated parameters by using data with no outliers. In the second case, parameters are estimated for the data having 10% contamination of the observations as outliers in the y-direction. The ME unfortunately gives poor results when outliers are in the X direction (Norazan,^15^). The results are obtained by averaging the 50,000 iterations of simulated data using a sample size of 100. This table shows that for case 1, all methods performed equally well when the data has no outlier. But for case II, the estimates provided by the proposed ME are very close to the values of the parameters from which the simulation was carried out. The results obtained from the considered estimator are also efficient, except for the OLS method. The OLS method again failed to provide reliable estimates when the data had outliers, which validated the results obtained in Section “Graphical comparison of the proposed RME with existing RME”. The proposed ME is also efficient for small sample sizes and can be used to save money, which is a major purpose of sampling and estimation.

## Conclusion

The results obtained in previous sections showed that the proposed RME is more general and efficient than the considered RME. The proposed ME showed that its behavior is exactly similar to other well-known RMEs, and the $\psi$-function given by the ME is more continuous before it redescends. The proposed estimator is very simple, more general, and flexible, and it converges very quickly as compared to previous MEs. The real-life data applications showed that the proposed RME is more efficient and has minimum SSE in the presence of outliers. It is also revealed from simulation studies that the values of coefficients obtained from the proposed robust estimator are very close to parameters and very similar to those of famous robust estimators such as Huber, Hample, Andrew, and Tukey. The proposed estimator can also be used to estimate the population mean using different sampling techniques for the data with outliers.

## Data Availability

The datasets used and/or analyzed during the current study are available from the corresponding author upon reasonable request. Further, no experiments on humans and/or the use of human tissue samples involved in this study.
